# GP-delivered brief weight loss interventions: a cohort study of patient responses and subsequent actions, using conversation analysis in UK primary care

**DOI:** 10.3399/bjgp18X698405

**Published:** 2018-08-14

**Authors:** Charlotte Albury, Elizabeth Stokoe, Sue Ziebland, Helena Webb, Paul Aveyard

**Affiliations:** Nuffield Department of Primary Care Health Sciences;; Loughborough University, Loughborough.; Nuffield Department of Primary Care Health Sciences;; Department of Computer Science, University of Oxford, Oxford.; Nuffield Department of Primary Care Health Sciences;

**Keywords:** conversation analysis, health behaviours, medical communication, primary health care

## Abstract

**Background:**

Guidelines encourage GPs to make brief opportunistic interventions to support weight loss. However, GPs fear that starting these discussions will lead to lengthy consultations. Recognising that patients are committed to take action could allow GPs to shorten brief interventions.

**Aim:**

To examine which patient responses indicated commitment to action, and the time saved if these had been recognised and the consultation closed sooner.

**Design and setting:**

A mixed-method cohort study of UK primary care patients participating in a trial of opportunistic weight management interventions.

**Method:**

Conversation analysis was applied to 226 consultation audiorecordings to identify types of responses from patients that indicated that an offer of referral to weight management was well received. Odds ratios (OR) were calculated to examine associations between response types and likelihood of weight management programme attendance.

**Results:**

Affirmative responses, for example ‘yes’, displayed no conversational evidence that the referral was well received and showed no association with attendance: ‘yes’ (OR 1.2, 95% confidence interval [CI] = 0.37 to 3.95, *P* = 0.97). However, ‘oh’-prefaced responses and marked positive responses, for example ‘lovely’, showed conversational evidence of enthusiasm and were associated with higher odds of commercial weight management service attendance. Recognising these could have saved doctors a mean of 31 seconds per consultation.

**Conclusion:**

When doctors make brief opportunistic interventions that incorporate the offer of help, ‘oh’-prefaced or marked positive responses indicate enthusiastic acceptance of the offer and a higher likelihood of take-up. Recognising these responses and moving swiftly to facilitate patient action would shorten the brief intervention in many cases.

## INTRODUCTION

National guidelines exhort GPs to use brief opportunistic interventions to encourage patients to improve their health behaviours.^1^^–^3 A recent trial showed that one such intervention for weight loss was acceptable to patients and doctors, and could effectively reduce population mean weight.^4^ Although brief interventions have been shown to be successful, GPs report several barriers to their frequent delivery in primary care consultations, citing limited consultation time as a key restriction.^5^^,^^6^ Healthcare professionals are often unsure whether, and how best, to raise the topic of their patients’ weight,^7^ and there is sparse evidence about how GPs can deliver effective interventions that are acceptable to patients and yet do not take up unnecessary consultation time. In this article the authors examine patient responses during the Brief Interventions for Weight Loss (BWeL) trial, where GPs intervened with consecutively attending patients who were overweight but not consulting for help with weight loss. The brief intervention comprised endorsing, offering, and facilitating a referral to a commercial weight management service (CWMS) free of charge. Following patient response to the offer of a free referral, GPs often entered into negotiation or further explanations, which lengthened consultations.

The aim of this study was to identify patterns of patient responses to the initial announcement of the offer of referral, which displayed that the offer had been well received by the patient, and which could be associated with future action. Conversation analysis (CA) was used to systematically explore and document observable patterns of communication. A leading principle of this method is that talk is systematic and orderly, and can be broken down into a series of actions.^8^ This enables identification of relationships between conversational patterns and specific outcomes.^9^ CA has been used to research medical interactions since the early 1980s, and extensive focus has been placed on communication in general practice.^9^^–^11 This approach highlights, for example, strategies that a doctor can use to avoid unnecessary prescriptions for antibiotics^12^ or to encourage patients to raise additional concerns.^13^ This method can be combined with statistical measures to further illustrate findings.^14^^,^^15^ In this study CA was combined with outcome data regarding CWMS attendance, enabling the researchers to explore relationships between patient responses to the offer of CWMS referral and future action. Early identification of responses that indicate an offer is well received by patients — and is associated with future action — could help GPs to tailor their discussions to avoid unnecessarily lengthy negotiations.

**How this fits in**

Guidelines encourage doctors to identify patients who are obese and refer them to weight management services. However, doctors fear that such conversations take a long time. In this study of consultation audiorecordings of doctors offering such referrals, it is shown that saying ‘yes’ to an offer does not indicate acceptance, but other responses including saying ‘oh yes’ do. Recognising these could allow doctors to shorten the consultation.

## METHOD

### Context and participants

In the BWeL trial, the researchers sought to enrol all patients attending 137 different GPs where the patient had a body mass index (BMI) ≥30 kg/m^2^ (or ≥25kg/m^2^ if Asian). Enrolment was from 4 June 2013 to 23 December 2014. Participating GPs received video-mediated training in intervention delivery but were not provided with a standardised script. At the end of the consultation, GPs in the intervention arm made an audiorecorded opportunistic brief intervention where they endorsed, offered, and facilitated a referral to a weight management service (either Slimming World^®^ or Rosemary Conley). Participants were telephoned after 3 months and seen in person after 12 months, where the researchers ascertained whether participants had tried to lose weight and the means by which they had had done this. Patient attendance data was provided by the weight management services.

Participants had the option to decline audiorecording, or to request deletion after the intervention had been delivered. Of 940 participants, half were randomly selected for audiorecording. Some GPs did not record, though prompted, or did not make an offer, some participants did not consent to be recorded, and some recordings were rendered unusable for technical reasons; consequently 226 recordings were available for this analysis. The recorder was visible to the GP and participant and was started when the GP was about to deliver the brief intervention.

### Data analysis

CA was conducted by one of the authors, who listened to the data and prepared specialist Jeffersonian transcripts, which highlighted information about the way talk was delivered. This included intonation, timing, and organisation of turns at talk.^16^ Audio data were analysed in conjunction with transcripts to identify how participants responded to the offer of a free referral, and how GPs reacted to participant responses. In line with the aim of identifying cues that indicate interventions do not need to be pursued further, analysis focused on the initial announcement of the offer of a free referral, and how participants responded at this early stage of the discussion. During CA the authors were blinded to patient attendance data, and displays that an offer had been positively received, or ‘positive reception displays’ (PRDs), were identified. This was done through attention to type of utterance, how and where they were produced, and the responses that they received. Where a pattern was identified through CA, statistical analysis was used to corroborate the study’s findings. The authors were interested in patient action following the intervention, so the outcome measure for this study was patient attendance at one or more CWMS session. Participant responses to the initial offer of CWMS referral were grouped into a series of utterance categories, and the authors then assessed on whether these categories were associated with attending the CWMS, calculating odds ratios (ORs) and 95% confidence intervals (CI) for the likelihood of attending relative to the most common response, which was ‘yeah’. Statistical analyses were conducted using OpenEpi (version 3.01).

## RESULTS

There were 19 different responses produced following the initial announcement of the offer of a free CWMS referral. Table 1 shows the breakdown of the utterance types most frequently produced in response to the GP’s initial announcement of the offer of a free CWMS referral. Although there was some variation in how referrals were offered, Box 1 demonstrates a typical consultation sequence where an announcement was followed by a response from the patient, and then an explicit offer from the GP, or further negotiation. The results presented in this article focus on patient responses to these initial announcements (line 5, Box 1). In accordance with convention of CA presentation in clinical journals, the transcripts have been simplified for presentation here. In these excerpts, a full stop in brackets represents a gap in talk of <0.3 seconds and a number in brackets indicates a timed gap in talk. A full stop at the end of turn represents falling intonation; a comma indicates a slight rise in intonation; and a question mark is used when intonation strongly rises.

Box 1. **Typical BWeL consultation sequence**

**Table:** 

**Line**	**Action**	**Speaker**	**Excerpt**
1	Announcement	DOC:	We’ve got the option to offer you well a good opportunity to offer you (0.4) erm the er chance to have fully (.) er funded on the NHS (.) er twelve er er so er Slimming World er (0.4) to help lose weight?
2			
3			
4			

5 →	Announcement response	PAT:	Oh right,

6	Explicit offer of referral	DOC:	Would you like to do that?

7	Response	PAT:	Er yeah?

8		DOC:	Yeah.
9	Confirmation check		Very good,
10	Assessment		There’s lot of health benefits to losing weight.
11	News delivery		And it’s er (0.6) it’s free,

12	Information receipt	PAT:	Yeah.

*BWeL = Brief Interventions for Weight Loss. DOC = doctor. PAT = patient. (Number) indicates gap in talk, seconds. (.) indicates gap in talk of* <*0.3 seconds. A full stop at end of turn represents falling intonation. Comma at end of turn indicates slight rise in intonation. ? indicates strong rise in intonation.* → *= line of significance.*

**Table 1.** Relationship between utterance and attendance across most common response types^a^ (*N* = 226)

**Utterance type**		**Example**		**CWMS attendance**	**Utterance, *n* (%)**	**Comparator, *n* (%)**	***P*-value**	**Odds ratio**	**95% CI**
1. Yeah	1	DOC:	What that means is that erm we can offer you erm erm basically a support programme to help with you losing weight.	+ −	19 23	n/a n/a	n/a n/a	1 (comparator)	N/A
	2								
	3								
	4								
	5								
	6								
	7								
	8								
	**9**	**PAT:**	**Yeah.**						

2. Oh-prefaced positive response	1	DOC:	It’s free (.) with this referral	+	16 (84)	19 (45)	0.008	6.27	1.68 to 30.48
	2								
	3								
	**4**	**PAT:**	**Oh right yeah I’ll try anything,**	−	3 (16)	23 (55)			

3. Right	1	DOC:	What they’re offering here is for you to go and be funded to join a group weight management session, one of the commercial companies.	+ −	8 (67) 4 (33)	19 (45) 23 (55)	0.33	2.38	0.62 to 10.34
	2								
	3								
	4								
	5								
	6								
	7								
	8								
	9								
	**10**	**PAT:**	**Right.**						

4. Yes	1	DOC:	We know the best way for you to lose weight is for you to go for a commercial weight management service. I can refer you now for free if you’d like.	+ −	8 (50) 8 (50)	19 (45) 23 (55)	0.97	1.21	0.37 to 3.95
	2								
	3								
	4								
	5								
	6								
	7								
	8								
	9								
	10								
	11								
	**12**	**PAT:**	**Yes.**						

5. Okay	1	DOC:	So (.) as part of this study er we’re able to offer you these sessions without any cost (.) [to yourself,	+ −	8 (40) 12 (60)	19 (45) 23 (55)	0.91	0.81	0.26 to 2.41
	2								
	3								
	4								
	5								
	6								
	7								
	8	PAT:	[Okay.						

6. No	1	DOC:	And we can give you a free voucher for this so you could freely take part in this (.) would you be interested in this.	+ −	2 (20) 16 (80)	19 (45) 23 (55)	0.02	0.16	0.02 to 0.69
	2								
	3								
	4								
	5								
	6								
	7								
	8								
	**9**	**PAT:**	**No.**						

7. Marked positive stance	1	DOC:	You get given twelve free sessions at uh Weight Watchers which I think is in Citychester Is that something that you’d quite fancy doing?	+ −	26 (79) 7 (21)	19 (45) 23 (55)	0.006	4.40	1.59 to 13.14
	2								
	3								
	4								
	5								
	6								
	7								
	8								
	9								
	**11**	**PAT:**	**Yes please.**						

8. Marked negative stance	1	DOC:	We know that the best way for you to lose weight is to go to a commercial weight management service (.) and I can refer you now for free if-if you like,	+ −	1 (14) 6 (86)	19 (45) 23 (55)	0.26	0.21	0.008 to 1.57
	2								
	3								
	4								
	5								
	6								
	7								
	8								
	9								
	10								
	11								
	**12**	**PAT:**	**I haven’t really erm been interested in it really.**						
	**13**								
	**14**								
	**15**								

*CWMS = commercial weight management service. DOC = doctor. N/A = not applicable. PAT = patient. (.) indicates gap in talk of* <*0.3 seconds. A full stop at end of turn represents falling intonation. Comma at end of turn indicates slight rise in intonation.[ indicates where talk is overlapping*.

a Only eight of the 19 different response types are explored in this study and presented in this article.

### Stand-alone affirmative responses

Negative responses to the announcement of a free referral, such as ‘no’ (utterance types 6 and 8, Table 1) were not identified as displays an offer had been well received, and were not associated with attendance at CWMS (20%, *n* = 2, attended versus 80%, *n* = 16, not attended, Fisher’s exact test *P* = 0.02). However, analysis showed that responses that appear to be affirmative, such as ‘yes’ and ‘yeah’ were not PRDs when produced as standalone utterances following the initial offer. GPs frequently treated these responses as displays that an offer was positively received, however they were often followed by further questions from a participant, or with a negative response later in the consultation. While affirmative responses may be expected to convey agreement, these utterances typically display *‘*acknowledgement’ of the initial offer, rather than conveying a stance to its acceptability.^17^ These analyses were corroborated by statistical results that showed no association between responses that were treated as a stand-alone affirmative response by a GP and subsequent attendance at the CWMS (for examples ‘yes’ 50%, *n* = 8, attended versus 50%, *n* = 8, not attended, Fisher’s exact test *P* = 0.97). These are shown in utterance types 1, 3, 4, and 5 in Table 1.

### Positive reception displays

This article focuses on two displays that an intervention was positively received, (utterances 2 and 7, Table 1). These occur early in the consultation following initial announcement of the offer of a free referral, shown in lines 1–4, Box 1.

#### Marked positive stance

One response type produced by participants following the announcement of the offer of a free CWMS referral was a ‘marked positive stance’, which displayed positivity when receiving or assessing an offer (utterance type 7, Table 1). These responses go beyond neutrally acknowledging the offer of free referral, and display some positive assessment or acknowledgement, such as ‘yes please’ or ‘lovely’, as in Box 2.

Box 2. **Display of marked positive stance**

**Table:** 

**Line**	**Action**	**Speaker**	**Excerpt**
1	Announcement	DOC:	So what this project is er offering people (.) if you’d like (.) er would be a twelve-week course through one of these commercial weight loss programmes paid for by the health service.
2			
3			

4→	Marked positive stance	PAT:	Lovely.

*DOC = doctor. PAT = patient. (.) indicates gap in talk of* <*0.3 seconds. A full stop at end of turn represents falling intonation.* → *= line of significance.*

The patient’s utterance of ‘lovely’ acts to display that the announcement of a free weight loss referral has been positively received. This response was typical of the types of marked positive stances produced by most participants, which could either be free standing, such as ‘lovely’ in Box 2, or with additional turn components. These responses were usually produced soon after the doctor had finished their turn, or in overlap with the doctor’s turn. They were also often higher in pitch than surrounding talk, a feature associated with receiving good news.^18^ This response type typically comprised of words that went beyond just acknowledgement, and conveyed the acceptability of the offer, although they often expressed varying levels of enthusiasm. A marked positive stance when a participant was informed of the news of a free referral, was commonly followed by displays of positivity throughout the remainder of the consultation, and acceptance of the offer of the free CWMS referral. A number of responses categorised as displaying a marked positive stance were prefaced with an acknowledgement token like ‘yes’ or ‘yeah’. However, these utterances were combined with other turn components to assert positive positioning. This utterance type was frequently responded to by GPs as a PRD, as they typically closed the consultation soon after this response, and rarely engaged in further negotiation (Box 3).

Box 3. **Moving to close following displays of marked positive stance**

**Table:** 

**Line**	**Action**	**Speaker**	**Excerpt**
1	Announcement	DOC:	Which means that er you get given twelve free sessions at er
2			Weight Watchers which I think is in Citychester.
3			Is that something that you’d quite fancy doing?

4→	Marked positive stance	PAT:	Yes please.

5	Close	DOC:	Excellent.
6			Well we’ll pass you forward to do that.

*DOC = doctor. PAT = patient. (Number) indicates gap in talk, seconds. (.) indicates gap in talk of* <*0.3 seconds. A full stop at end of turn represents falling intonation. Comma at end of turn indicates slight rise in intonation. ? indicates strong rise in intonation.* → *= line of significance.*

Doctors who recognised a marked positive stance typically took ≤9 seconds to close a consultation.

**Figure 1.**
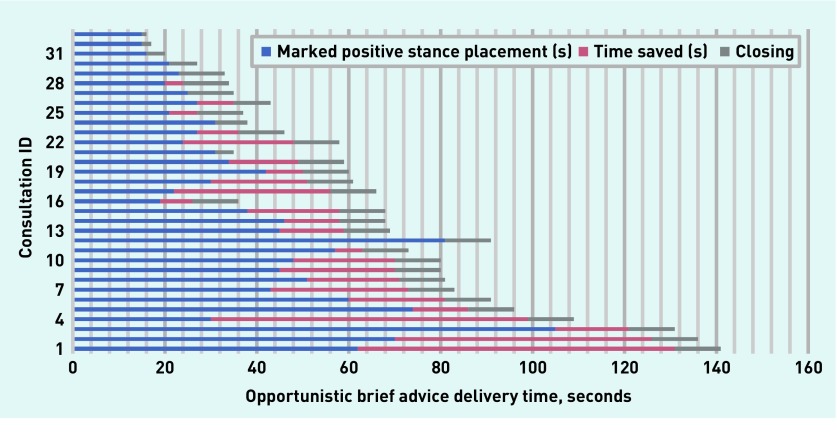
***Placement and timing of marked positive stance within the consultation. Each ‘Consultation ID’ number represents a patient within this group. The number assigned is an arbitrary one which does not represent the sequence in which these consultations were delivered.***

Statistical analysis showed a significant relationship between a marked positive stance and subsequent attendance at the CWMS (79%, *n* = 26, attended versus 21%, *n* = 7, not attended, Fisher’s exact test *P* = 0.006). The mean time for these discussions was 64 seconds (range 16–141 seconds), while a marked positive stance was produced at a mean of 39 seconds (range 15–105 seconds) (Figure 1). Recognising these utterances as PRDs, and moving to close, could have saved a mean of 16 seconds per consultation.

#### Turn initial ‘Oh’ receipts

Following announcement of the offer of a free CWMS referral some participants displayed a turn initial ‘oh’ receipt (utterance type 2, Table 1). These are responses where ‘oh’ is either a standalone utterance or prefaces an acknowledging response or marked positive stance, for example ‘Oh right’ or ‘Oh definitely’. In Box 4 the GP formulates a treatment recommendation, and the patient responds with a turn initial ‘oh’ receipt at line 4. The patient says ‘oh’ immediately following the news of free referral, and this utterance is higher in pitch than this patient’s prior talk. This is not responded to by the doctor as a PRD, as they enter into further explanation, and re-formulate their offer in line 15.

Box 4. **Turn initial ‘Oh’ receipt**

**Table:** 

**Line**	**Action**	**Speaker**	**Excerpt**
1	Announcement	DOC:	It would be possible to go and see s- erm (.) one of these have you come across them, Rosemary Conley and the um that type of thing- (.) of weight loss things.
2			
3			

4 →	Announcement response	PAT:	Oh right,
5			Okay.

6	Information delivery	DOC:	Where you go to various classes (.) basically.

7	Information receipt	PAT:	Oh right.

8	Account	DOC:	Because your weight is a bit high,

9	Continuer	PAT:	Yeah.

10	Information delivery	DOC:	And it would be very helpful for you to actually lose some weight.

11	Information receipt	PAT:	Okey dokey.

12	Explicit offer of referral	DOC:	That um they’re suggesting that you might want to go and take that up and go and see them,
13			
14			I don’t know if you’d be interested in that.

*DOC = doctor. PAT = patient. (.) indicates gap in talk of* <*0.3 seconds. A full stop at end of turn represents falling intonation. Comma at end of turn indicates slight rise in intonation.* → *= line of significance.*

**Figure 2.**
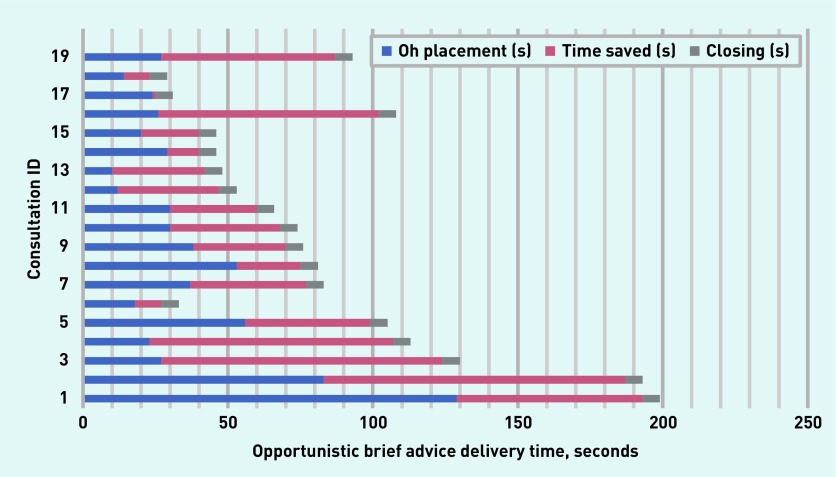
***Placement and timing of turn initial ‘Oh’ responses within the consultation. Each ‘Consultation ID’ number represents a patient within this group. The number assigned is an arbitrary one which does not represent the sequence in which these consultations were delivered.***

An ‘Oh’, in response to information can show that it is news.^19^ In the current study data ‘Oh’ in this position was consistently followed by positive responses in that participant’s subsequent turns, and by participant agreement to the recommended treatment. Similar to a marked positive stance, an ‘Oh’ often occurred in overlap with the doctor’s talk and was higher in pitch than the patient’s prior talk. Therefore, these ‘Oh’- prefaced responses were identified as PRDs. Statistical analysis supported the findings (utterance 2, Table 1), and demonstrated an association between ‘Oh’-prefaced responses and participant attendance at a weight loss service (84%, *n* = 16, attended versus 16%, *n* = 3 not attended, Fisher’s exact test *P* = 0.008). Despite the positive characteristics of this utterance, doctors infrequently treated an ‘Oh’ response as indicative of positive reception. When they did, however, they were able to successfully close a consultation in ≤6 seconds without further discussion. The mean length of these discussions was 87 seconds (range 29–199 seconds) with ‘Oh’ occurring at a mean of 36 seconds (range 10–129 seconds) (Figure 2). Recognising ‘Oh’ as indicating future participant action regarding CWMS attendance, and providing a sign that closing may be initiated, could have saved an average of 45 seconds of brief advice discussion.

## DISCUSSION

### Summary

The authors have shown that brief interventions in primary care can be very brief when GPs attend to specific reception displays. Understandably, GPs frequently attended to ‘yes’ or ‘yeah’ as positive responses to the announcement of the offer of a free CWMS referral, yet the authors observed that such responses neither acted as positive reception displays within the consultation, nor were they associated with subsequent attendance at the CWMS. Therefore, these patients may require more information from their GP. The authors identified two types of participant response (displays of marked positive stance and turn initial ‘Oh’ receipts), which displayed that an offer was positively received. Statistical analysis demonstrated an association between these responses and future action, and established that in-consultation PRDs were associated with participant attendance at weight management services. Displays of marked positive stance and ‘Oh’-prefaced responses occurred early in the discussion. Recognising these could have saved a mean of 31 seconds per consultation.

### Strengths and limitations

A strength of this study was the use of audiorecorded data to identify exact participant responses to a brief opportunistic intervention and the time at which they were produced. Data were collected across a number of general practices and from diverse patient groups. It is relatively unusual for CA to combine primary care consultation data with reliable information about what happened next: in this case the researchers were able to make associations between real-time conversations and subsequent attendance at CWMS. A limitation is that audiorecorders were visible to both patient and GP, and this may have influenced discussions. Without video data it is possible that some gestures that indicate positive reception were not identified. The authors would recommend use of video recordings (increasingly available through archives such as ‘One in a Million’)^20^ in future studies to complement this analysis of audiorecordings.

### Comparison with existing literature

Previous CA of recorded brief interventions (to promote smoking cessation) have identified several ways in which clinicians can make effective brief interventions.^21^^,^ 22 However, this is, to the authors’ knowledge, the first time that behavioural advice has been associated with a patient’s next action regarding CWMS attendance. The current results build on other conversation analytic work, which identified ‘Oh’ as indicating prior news has been good,^19^ and foreshadowing future acceptance of advice,^17^ by also identifying associations between ‘Oh’ and subsequent action.

### Implications for practice

The GP consultation is tightly time limited raising concerns about how even a brief opportunistic intervention can be included. This analysis shows that, through attending to clues in what patients say, doctors can shorten delivery significantly. When delivering a brief intervention for weight loss, doctors can attend to displays of marked positive stance, and turn initial ‘Oh’ receipts. They occur early in the consultation and are clearly associated with uptake and attendance. Attending to these clues can avoid unnecessary negotiations and significantly shorten the delivery time of brief interventions for weight loss, thereby addressing one of the main concerns GPs report about intervening to change behaviour.
